# Supplementary material for: Graph coloring using the reduced quantum genetic algorithm
Source: PeerJ Comput Sci. 2022 Jan 3;8:e836. doi: 10.7717/peerj-cs.836 (PMC8771768; doi:10.7717/peerj-cs.836)
Supplement: Supplemental Information 1 [file peerj-cs-08-836-s001.pdf]

Graph Coloring Using the Reduced Quantum Genetic Algorithm

(Supplementary Information)

Sebastian Mihai Ardelean, Mihai Udrescu

1 Additional experimental results

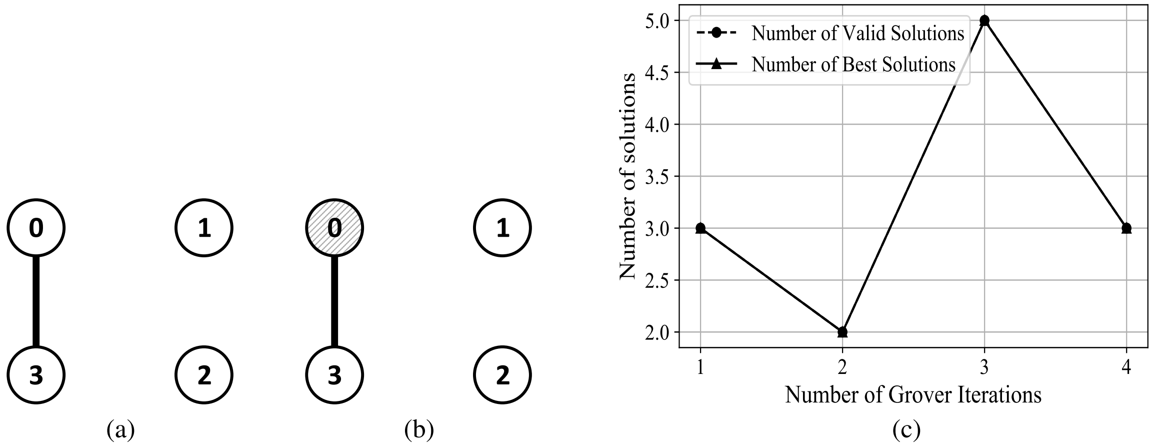

Figure S1: Panel a) shows the Erdős-Rényi graph generated with edge probability 0.4 and 4 nodes, which we used for the coloring problem. Panel b) shows the same graph colored with the solution found by our algorithm. Nodes 1, 2, and 3 have the same color and node 0 is colored differently. The best result for this graph uses only 2 colors.The chromatic number for this graph is 2 and the algorithm, even if it is configured to use 3 colors, it determined that the best solution for coloring the graph is by using only 2 colors, thus determining both the coloring and the chromatic number. Panel c) depicts the experimental results; in each iteration the algorithm found only best solutions.

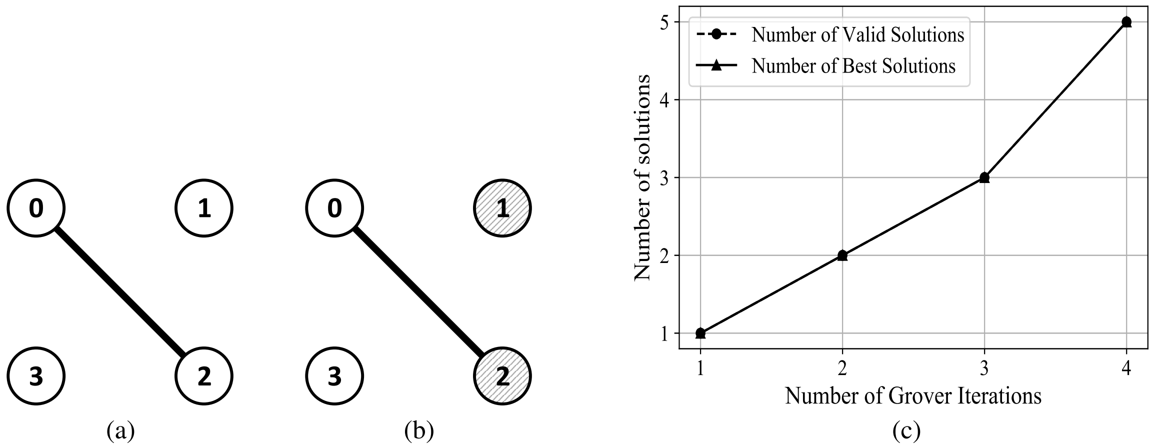

Figure S2: Panel a) shows an Erdős-Rényi graph generated with an edge probability of 0.4 and 4 nodes, which we used for coloring problem. Panel b) shows the same graph colored with the solution found by the algorithm. The result for this graph uses only 2 colors, so that nodes 1 and 2 share one color while nodes 0 and 3 are colored using the second one. The chromatic number for this graph is 2 and the algorithm, even if it is configured to use 3 colors, it determined that the best solution for coloring the graph is by using only 2 colors, thus determining both the coloring and the chromatic number. Panel c) depicts the experimental results, in each iteration, the algorithm found only best solutions with a maximum of 5 solutions found after 4 iterations.

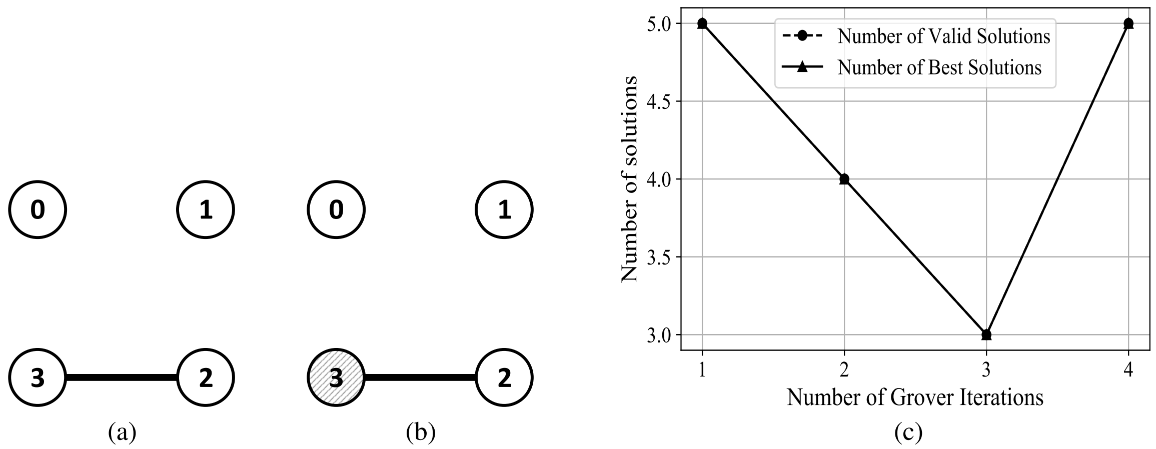

Figure S3: Panel a) shows the Erdős-Rényi graph generated with edge probability 0.4 and 4 nodes, which we used for the coloring problem. Panel b) shows the same graph colored with the solution found by our algorithm. The result for this graph uses only 2 colors: nodes 0, 1 and 2 have the same color and node 3 is colored differently. The chromatic number for this graph is 2 and the algorithm, even if it is configured to use 3 colors, it determined that the best solution for coloring the graph is by using only 2 colors, thus determining both the coloring and the chromatic number. Panel c) presents the experimental results; in each iteration the algorithm found only best solutions, with a maximum of 5 solutions found in iterations 1 and 4.

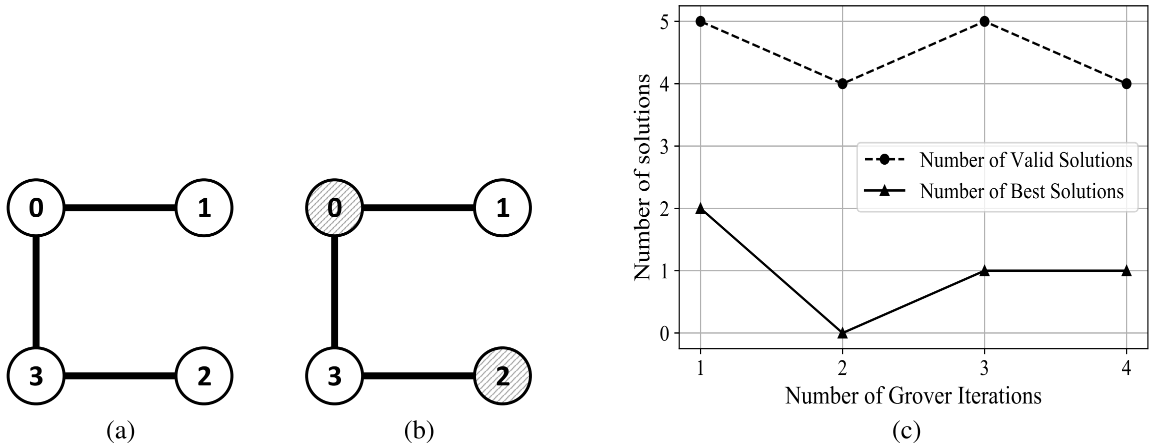

Figure S4: Panel a) shows an Erdős-Rényi graph generated with edge probability 0.4 and 4 nodes, which we used for the coloring problem. Panel b) shows the same graph colored with the solution found by our algorithm. The result for this graph uses only 2 colors, so that nodes 1 and 3 are colored using first color and nodes 0 and 2 are colored using the second one. The chromatic number for this graph is 2 and the algorithm, even if it is configured to use 3 colors, it determined that the best solution for coloring the graph is by using only 2 colors, thus determining both the coloring and the chromatic number. Panel c) depicts the experimental results; after 1 Grover iteration the algorithm produced 5 valid solutions from which 2 are best solutions.

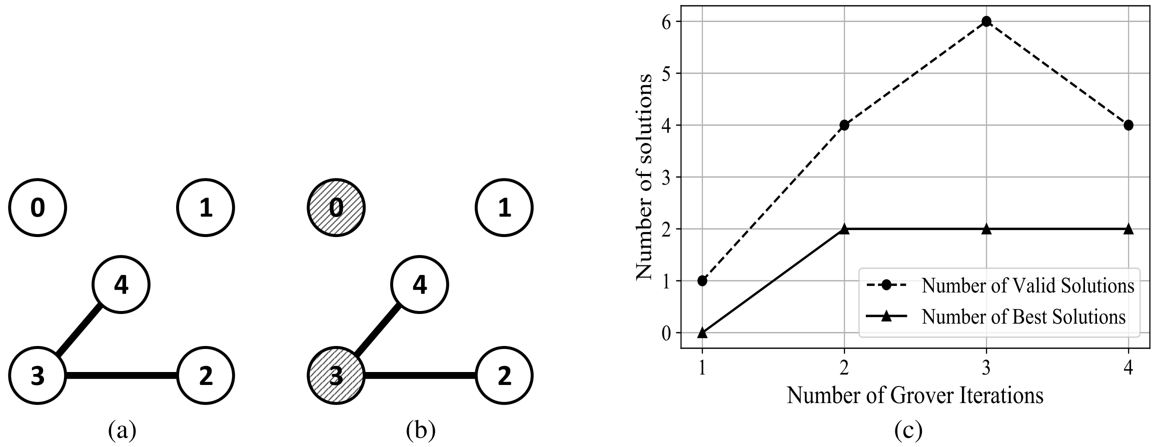

Figure S5: Panel a) shows an Erdős-Rényi graph generated with edge probability 0.4 and 5 nodes, which we used for the coloring problem. Panel b) shows the same graph colored with the solution found by our algorithm. The solution for this graph uses 2 colors, so that nodes 0 and 3 are colored using the first color, and nodes 1, 2 and 4 are colored using the second one. The chromatic number for this graph is 2 and the algorithm, even if it is configured to use 3 colors, it determined that the best solution for coloring the graph is by using only 2 colors, thus determining both the coloring and the chromatic number. Panel c) depicts the experimental results: after 3 iterations the algorithm produced 6 valid solutions from which 2 are best solutions.

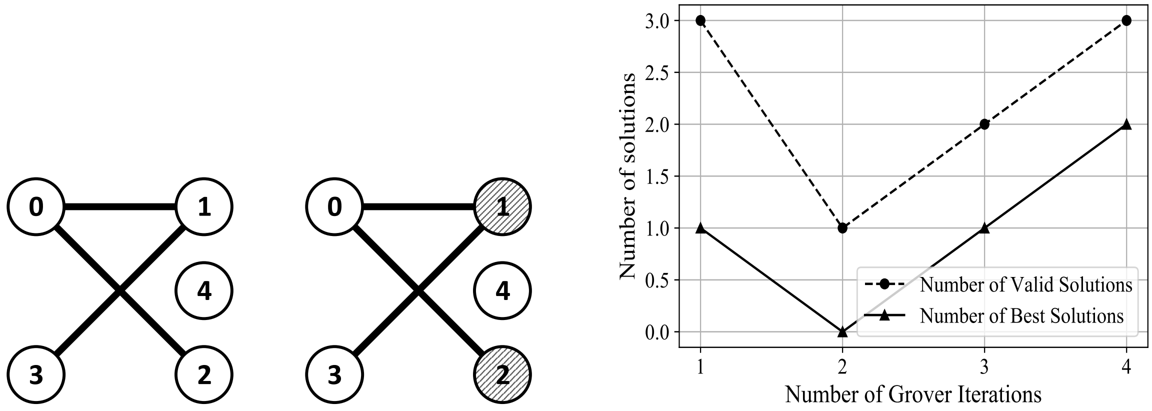

(a) (b) (c)

Figure S6: Panel a) shows an Erdős-Rényi graph generated with edge probability 0.4 and 5 nodes, which we used for the coloring problem. Panel b) shows the same graph colored with the solution found by our algorithm. The solution for this graph uses 2 colors, so that nodes 0, 3 and 4 are colored using the first color and nodes 1 and 2 are colored using the second one. The chromatic number for this graph is 2 and the algorithm, even if it is configured to use 3 colors, it determined that the best solution for coloring the graph is by using only 2 colors, thus determining both the coloring and the chromatic number. Panel c) depicts the experimental results; after 4 iterations the algorithm produced 3 valid solutions from which 2 are best solutions.

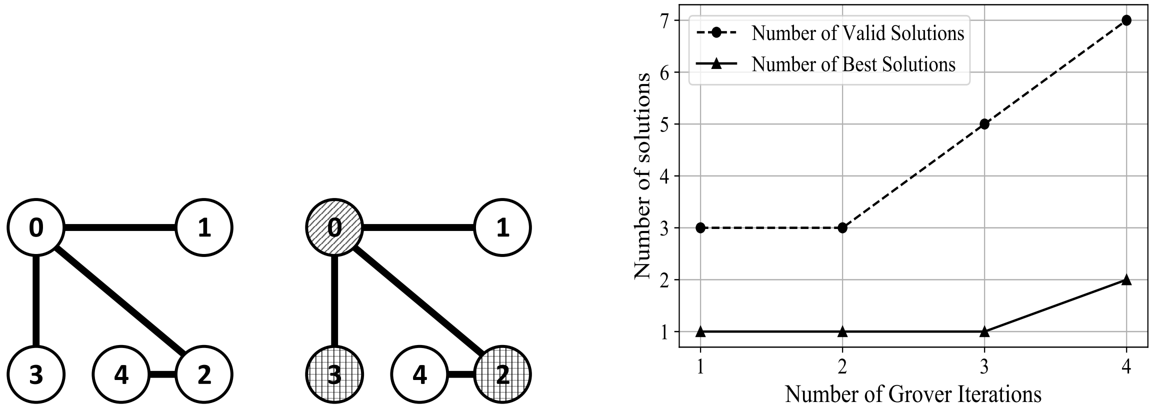

(a) (b) (c)

Figure S7: Panel a) shows an Erdős-Rényi graph generated with edge probability 0.4 and 5 nodes, which we used for the coloring problem. Panel b) shows the same graph colored with the solution found by our algorithm. The solution for this graph uses 3 colors, so that nodes 3 and 2 are colored using the first color; nodes 1 and 4 are colored using the second color; node 0 is colored using the third color. Panel c) depicts the experimental results: after 4 iterations, the algorithm produced 7 valid solutions from which 2 are best solutions.

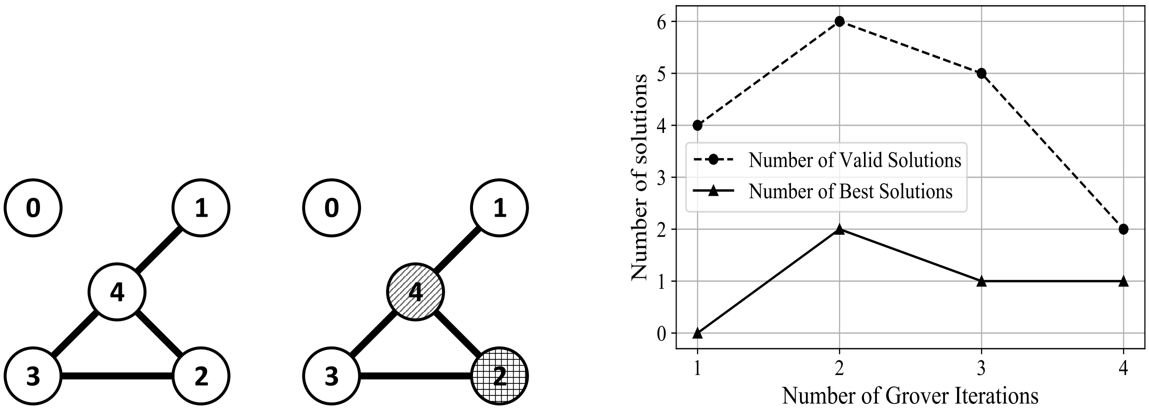

(a) (b) (c)

Figure S8: Panel a) shows an Erdős-Rényi graph generated with edge probability 0.4 and 5 nodes, which we used for the coloring problem. Panel b) shows the same graph colored with the solution found by our algorithm. The solution for this graph uses 3 colors, so that nodes 0, 1 and 3 are colored using the same color and nodes 2 and 4 are colored using different colors. Panel c) depicts the experimental results: after 2 iterations, the algorithm produced 6 valid solutions from which 2 are best solutions.

2 Graph coloring example

In this section, we present an example of how the Reduced Quantum Genetic Algorithm is applied to a typical genetic search problem.

Problem: Considering the graph in Figure S9 and a number of 3 colors, find a way of coloring the nodes such that no two adjacent nodes are colored with the same color.

Solution: In a classical genetic approach, we start by generating a population with each chromosome encoding 4 genes, each gene representing a color. In the quantum version, the chromosome encoding requires 8 qubits. Each color is represented with 2 qubits, thus we will consider the combination 11 as an invalid color. The chromosome will encode valid and invalid colors as superposed basis states, a chromosome that contains at least one invalid color is considered an invalid solution, as presented in Table 1.

We start with the initial state

$$
\begin{aligned}
|\psi\rangle_1 &= \frac{1}{16}\sum_{u=0}^{255}|u\rangle_i \otimes |0\rangle_i \\
&= \frac{1}{16}\begin{pmatrix} & |00000000\rangle & + & |00000001\rangle \\ + & |00000010\rangle & + & |00000011\rangle \\ + & \dots & & \\ + & |00010000\rangle & + & |00010001\rangle \\ + & |11111110\rangle & + & |11111111\rangle \end{pmatrix} \otimes |000000000\rangle .
\end{aligned} \tag{1}
$$

In Table 1 we present the chromosome encoding on 8-qubit classical values in superposition with valid and invalid flags.

We apply the fitness function defined in Section Implementation over the individual registers $|u\rangle_i$ obtaining the state presented in (2).

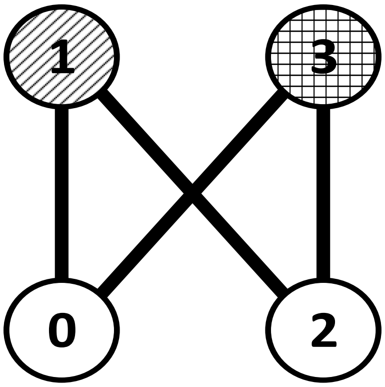

Figure S9: Randomly generated Erdős-Rényi graph with an edge probability of 0.7.

N_0	N_1	N_2	N_3	Validity
00	00	00	00	Valid
00	00	00	01	Valid
00	00	00	10	Valid
00	00	00	11	Invalid
⋮	⋮	⋮	⋮	⋮
11	11	11	10	Invalid
11	11	11	11	Invalid

Table 1: Chromosome binary configurations with valid and invalid flags.

$$|\psi\rangle_2 = |u\rangle_i \otimes |fitness_u\rangle_i \quad = \frac{1}{16}\begin{pmatrix} & |00000000\rangle & \otimes|100000000\rangle \\ + & |00000001\rangle & \otimes|100000010\rangle \\ + & |00000010\rangle & \otimes|100000010\rangle \\ + & |00000011\rangle & \otimes|011111111\rangle \\ + & \dots & \\ + & |00010000\rangle & \otimes|100000010\rangle \\ + & |00010001\rangle & \otimes|100000100\rangle \\ + & \dots & \\ + & |11111110\rangle & \otimes|011111111\rangle \\ + & |11111111\rangle & \otimes|011111111\rangle \end{pmatrix} \tag{2}$$

All least significant 8 bits of the fitness values represent two's complement numbers and the 9th bit represents the validity of the individual, 0 indicates an invalid individual, while 1 indicates a valid one.

The next step is to apply the oracle over the fitness register. We count the number of edges in graph—in this example is 4—and apply the oracle implemented as in Figure 4. In Equation (3) $|\psi\rangle_3^1$ is the pair register state after subtracting the number of edges from the fitness value and applying phase-shift. The pair register state after applying the Oracle is presented in Equation (4).

$$|\psi\rangle_3^1 = |u\rangle_i \otimes |fitness_u\rangle_i \quad = \frac{1}{16}\begin{pmatrix} & |00000000\rangle & \otimes|111111100\rangle \\ + & |00000001\rangle & \otimes|111111110\rangle \\ + & |00000010\rangle & \otimes|111111110\rangle \\ + & |00000011\rangle & \otimes|011111011\rangle \\ + & \dots & \\ + & |00010000\rangle & \otimes|111111110\rangle \\ - & |00010001\rangle & \otimes|100000000\rangle \\ + & \dots & \\ + & |11111110\rangle & \otimes|011111011\rangle \\ + & |11111111\rangle & \otimes|011111011\rangle \end{pmatrix} \tag{3}$$

$$|\psi\rangle_3^2 = |u\rangle_i \otimes |fitness_u\rangle_i \quad = \frac{1}{16}\begin{pmatrix} & |00000000\rangle & \otimes|100000000\rangle \\ + & |00000001\rangle & \otimes|100000010\rangle \\ + & |00000010\rangle & \otimes|100000010\rangle \\ + & |00000011\rangle & \otimes|011111111\rangle \\ + & \dots & \\ + & |00010000\rangle & \otimes|100000010\rangle \\ - & |00010001\rangle & \otimes|100000100\rangle \\ + & \dots & \\ + & |11111110\rangle & \otimes|011111111\rangle \\ + & |11111111\rangle & \otimes|011111111\rangle \end{pmatrix} \tag{4}$$

We apply the diffuser operation over the fitness register from $|\psi\rangle_3^2$ and we get the state $|\psi\rangle_4$ as presented in Equation (5), with amplitudes $\alpha_0, \alpha_1, \alpha_2, \alpha_3, \dots, \alpha_{16}, \alpha_{19}, \alpha 20, \dots, \alpha_{255} \approx 0$ and $|\alpha_{17}|^2 + |\alpha_{18}|^2 = 1$.

$$|\psi\rangle_4 = |u\rangle_i \otimes |fitness_u\rangle_i \quad = \frac{1}{16}\begin{pmatrix} & \alpha_0\,|00000000\rangle & \otimes|100000000\rangle \\ + & \alpha_1\,|00000001\rangle & \otimes|100000010\rangle \\ + & \alpha_2\,|00000010\rangle & \otimes|100000010\rangle \\ + & \alpha_3\,|00000011\rangle & \otimes|011111111\rangle \\ + & \dots & \\ + & \alpha_{16}\,|00010000\rangle & \otimes|100000010\rangle \\ - & \alpha_{17}\,|00010001\rangle & \otimes|100000100\rangle \\ + & \dots & \\ + & \alpha_{254}\,|11111110\rangle & \otimes|011111111\rangle \\ + & \alpha_{255}\,|11111111\rangle & \otimes|011111111\rangle \end{pmatrix} \tag{5}$$

Having the highest fitness value, if we measure the individual register of $|\psi\rangle_4$ we obtain the corresponding individual (or one of the corresponding individuals, if there are more solutions). We get, with a high probability, one of the following basis states: $|00010001\rangle, |00010010\rangle$. Assuming that $|00010001\rangle$ is measured, we update the *max* value with the corresponding fitness value, and repeat the steps above until the *max* is not improved.

Suppose that, after applying Grover iterations, we measure $|00010010\rangle$ in individual register of $|\psi\rangle_{m-1}$, then in fitness register we will have $|100000100\rangle$. Since *max* value is not improved, we have the solution for our problem.

3 Circuit implementation

Listing 1: Quantum registers initialization

```
ind_qreg = QuantumRegister(ind_qreg_size,
    "ireg")
fit_qreg = QuantumRegister(fit_qreg_size + 1
    , "freg")
carry_qreg = QuantumRegister(carry_size,
    "carryreg")
oracle = QuantumRegister(oracle_ws_size,
    "oreg")
pos_no_of_edges = QuantumRegister(fit_qreg_size,"pedgesreg")
creg = ClassicalRegister(ind_qreg_size,"reg")
qc = QuantumCircuit(ind_qreg,
                fit_qreg,
                carry_qreg,
                oracle_ws,
                neg_no_of_edges,
                pos_no_of_edges,
                creg)
qc.h(ind_qreg)
qc.h(oracle_ws)
```

In Listing 1 we present the initialization step of the algorithm. In this step the quantum registers are initialized, the circuit is created and the individual quantum register and the oracle qubit are put in superposition. The individual quantum register contains the color combinations for solving the graph coloring and fitness quantum register is used for storing the fitness value. The carry quantum register is used in the oracle for performing the subtraction and addition. The quantum register named in Listing 1 as *pos_no_of_edges* stores the number of edges in the graph and is used in the oracle. This value is used along with the fitness value to perform the subtraction and addition.

Listing 2: Appending U_{fit} Sub-circuit

```
qc.append(ufit_instr,
    [ind_qreg[q] for q in range(0,ind_qreg_size)]+
    [fit_qreg[q] for q in range(0,fit_qreg_size+1)]
               )
```

Listing 2 shows the append of U_{fit} subcircuit to the circuit with individual and fitness quantum registers as inputs. The implementation of U_{fit} subcircuit is presented in Algorithm 2.

Listing 3: Appending Oracle Sub-circuit

```
qc.append(oracle_instr,
   [pos_no_of_edges_qreg[q] for q in range(0,fit_qreg_size)]
   +[fit_qreg[q] for q in range(0,fit_qreg_size)]
   +[oracle[0],carry_qreg[0],carry_qreg[1]]
               )
```

The oracle subcircuit implementation is presented in Algorithm 3 while the subcircuit usage is presented in Listing 3. As it can be observed, *pos_no_of_edges*, fitness and oracle quantum registers

are used as an input to the subcircuit. In Listing 4 the appending of Grover diffuser subcircuit is presented. The implementation for this subcircuit is provided in Figure 6 and is applied to the fitness quantum register, valid and oracle qubits.

Listing 4: Appending Grover Diffuser Sub-circuit

```
qc.append(grover_iter_inst,
    [fit_qreg[q] for q in range(0,fit_qreg_size+1)]
    +[oracle[0]])
```

The full code of our project is available on Github https://github.com/sebastianardelean/graphcoloringusingrqga.

Acronyms

RQGA Reduced Quantum Genetic Algorithm. 8

References
